# CBD Promotes Structural and Functional Epithelial Restoration and Alleviates Inflammation in a Mouse Model of Interstitial Cystitis

**DOI:** 10.3390/pharmaceutics18040458

**Published:** 2026-04-09

**Authors:** Dominika Peskar, Mojca Kerec Kos, Špela Tavčar, Katja Lakota, Nika Kojc, Peter Veranič, Andreja Erman

**Affiliations:** 1 Institute of Cell Biology, Faculty of Medicine, University of Ljubljana, 1000 Ljubljana, Sloveniaspela.bernard@gmail.com (Š.T.); peter.veranic@mf.uni-lj.si (P.V.); 2 Department of Biopharmacy and Pharmacokinetics, Faculty of Pharmacy, University of Ljubljana, 1000 Ljubljana, Slovenia; mojca.kerec-kos@ffa.uni-lj.si; 3 Department of Rheumatology, University Medical Centre Ljubljana, 1000 Ljubljana, Slovenia; katja.lakota@kclj.si; 4 Institute of Pathology, Faculty of Medicine, University of Ljubljana, 1000 Ljubljana, Slovenia; nika.kojc@mf.uni-lj.si

**Keywords:** urinary bladder, urothelium, permeability barrier, inflammation, interstitial cystitis, cannabidiol, cannabinoid receptors

## Abstract

**Background**: Interstitial cystitis (IC) is a debilitating lower urinary tract condition characterised by chronic inflammation of the bladder. As the aetiology remains unknown, current treatments are symptomatic, aiming to reduce inflammation and pain. Cannabidiol (CBD), the most common cannabinoid in industrial *Cannabis sativa* (hemp), is one of the most important pharmacologically active cannabinoids used in medicine due to its anti-inflammatory and antioxidant effects without psychoactive properties. While other cannabinoids have shown beneficial effects in animal models of IC, the impact of CBD on the urinary bladder and overall animal well-being has not been elucidated. **Methods**: Using a cyclophosphamide (CYP)-induced mouse model of IC, we investigated the effects of intraperitoneally administered CBD on bladder structure, function, inflammation, and animal behaviour. A multimodal approach was applied, including light and electron microscopy, immunolabeling, qPCR, transepithelial electrical resistance (TEER) measurements, behavioural testing, and monitoring of animals. **Results**: CBD treatment promoted the restoration of damaged urothelial structure and improved the integrity of the blood–urine barrier. Additionally, CBD exerted an anti-inflammatory effect, reducing oedema and infiltration of inflammatory cells in the bladder wall with chronic cystitis. Finally, the increased burrowing activity of CBD-treated mice suggests a benefit of CBD on overall well-being. **Conclusions**: Our findings suggest that CBD has a beneficial effect on the inflamed urinary bladder and could potentially serve as an adjunct treatment for patients with IC in the future.

## 1. Introduction

Interstitial cystitis (IC) is a chronic inflammatory condition characterised by pelvic pain and pressure, or discomfort associated with the urinary bladder, along with urinary symptoms such as urgency and frequency, without identifiable pathology. The incidence of IC is estimated at 15 per 100,000 people per year, with predominantly female populations affected [1,2]. This disorder is particularly complex due to its multifaceted nature and varying presentations, which can significantly impair the quality of life of affected individuals [3]. Due to the complexities of diagnosis, IC remains underdiagnosed. The precise aetiology of IC remains largely unknown, although several hypotheses have been suggested, including urothelial barrier dysfunction, neurogenic inflammation, and potential autoimmunity [4]. The clinical definition of IC has evolved, with different types categorised based on cystoscopic findings, particularly the presence of Hunner lesions in some patients [5].

The management of IC has historically included a range of interventions, from oral medications to intravesical therapies, focusing on symptom relief [6]. Recently, endogenous and plant-derived cannabinoids, particularly cannabidiol (CBD), a non-psychoactive component of hemp *(Cannabis sativa* L.) extract, have attracted attention as adjunctive therapeutics due to their significant analgesic and anti-inflammatory properties [7]. CBD acts by interacting with several receptors, such as the GPR5 receptor, various TRP receptors, 5-HT_1A_, PPARγ, and D2 [8], with lower affinity also for the type 1 cannabinoid receptor (CB1R) and type 2 cannabinoid receptor (CB2R), as a negative allosteric binder and inverse agonist, respectively [9,10,11,12]. CB1 receptors are expressed in the central and peripheral nervous systems as well as in gastrointestinal organs, while CB2 receptors are present mainly in peripheral tissues, particularly in immune tissues such as spleen, tonsils, thymus, and mast cells [13].

CB1R and CB2R have also been identified in the urinary bladder, particularly in the urothelium and nerve terminals [14,15,16,17]. The urinary bladder is a hollow organ, the lumen of which is lined by the epithelium known as the urothelium. It consists of undifferentiated basal cells, partially differentiated intermediate cells, and highly differentiated superficial cells that are in contact with urine. Due to the specialised apical plasma membrane and numerous well-developed tight junctions of the superficial cells, the urothelium functions as a high-resistance permeability barrier [18,19,20]. The urothelium is considered a mitotically quiescent epithelium with a very low cell turnover rate, limited to basal and intermediate cells under normal physiological conditions [21,22]. However, it responds rapidly to injury with intense proliferation to restore its damaged structure [23,24].

A growing body of literature demonstrates the positive anti-inflammatory and anti-oxidative effects of various cannabinoid CB1 and CB2 receptor ligands as a treatment approach in animal models of IC [15,25,26,27,28]. However, only recently has attention been given to the mechanisms of action of CBD on the inflamed urinary bladder to explore the potential role of CBD as a therapeutic agent in models of IC [29,30,31].

In this study, the effects of CBD were investigated in a mouse model of IC. The bladder-centric model of IC was established by repeated administration of low doses of cyclophosphamide (CYP) and systemic daily administration of CBD as a potential therapeutic intervention. Overall, the beneficial effects of CBD were observed at several levels: on damaged urothelial structure, compromised bladder barrier function, chronic bladder inflammation, and the well-being of the experimental animals.

## 2. Materials and Methods

### 2.1. Animals and Experimental Design

**Scheme 1:**
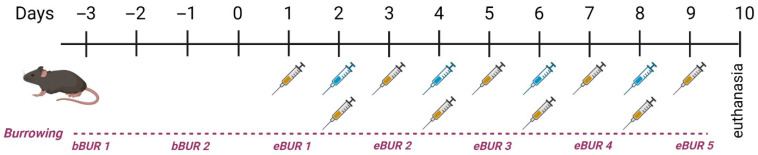
Graphical presentation of the experimental design. Blue syringes indicate CYP; yellow syringes indicate animal treatment (CBD or vehicle). bBUR: basal burrowing measurement; eBUR: experimental burrowing measurement. Created with Biorender. Erman, A. (2026) https://BioRender.com/vs3fc7c (accessed on 1 April 2026).

All animal experiments were performed in accordance with the Administration of the Republic of Slovenia for Food Safety, the Veterinary Sector, and Plant Protection, permit number U34401-4/2020/10. Fifty-five female C57BL/6 mice, aged 12–14 weeks and weighing 20–30 g, were included in the study across eight separate experiments. The animals were housed in polyacrylamide cages in groups of five under constant humidity (55%) and temperature (22 °C) with a 12/12 h light cycle. Water and food were available ad libitum. Before the experiment, the animals underwent a 7-day acclimatisation period. Animals were randomly divided into four groups: control (Ctrl; *n* = 10), cyclophosphamide (CYP)-treated group (*n* = 15), CYP and vehicle-treated group (CYP + veh; *n* = 15), and CYP and CBD-treated group (CYP + CBD; *n* = 15). In the three treatment groups, aseptic cystitis was induced as previously described [32]. Briefly, the animals received intraperitoneal (i.p.) injections of 80 mg/kg CYP (Sigma-Aldrich, Merck, Darmstadt, Germany) diluted in sterile saline four times during the experiment (on days 2, 4, 6, and 8), while the Ctrl group received a corresponding volume of sterile saline. Animals in the intervention groups (CBD and vehicle) received i.p. injections of either 1.5 mg/kg CBD (CBD group) or a corresponding volume of vehicle daily for nine days (days 1–9). Cannabidiol or CBD (2-[1R-3-methyl-6R-(1-methylethenyl)-2-cyclohexen-1-yl]-5-pentyl-1,3-benzenediol; Cayman Chemical, Ann Arbor, MI, USA), reconstituted in ethanol was diluted in Cremophor^®^ EL (castor oil polyoxyethylene ether, Merck, Darmstadt, Germany) and sterile saline in a 1:1:18 ratio [29]. The same mixture, without CBD, served as the vehicle. During administration, extreme care was taken to alternate the injection sites for each injection (alternating between the areas left and right of the linea alba). The general clinical condition and body weight of the animals were monitored throughout the experiment. Euthanasia was conducted on day 10. The experimental design is summarised in Scheme 1.

### 2.2. Preparation of Paraffin Sections and Cryosections of Urinary Bladder Tissue

For paraffin sections, excised urinary bladders were cut in half and fixed in 10% buffered formalin at 4 °C for 24 h, rinsed overnight with PBS, then dehydrated through a graded ethanol series (50–100%, 15 min each), cleared in xylene, and embedded in paraffin. The embedded tissues were sectioned at 5 µm thickness and stained with hematoxylin and eosin (H&E) or immunolabeled. The histological slides were examined with a brightfield microscope Nikon Eclipse E200 (Nikon, Amsterdam, The Netherlands).

For cryosections, the bladder halves were fixed in 3% paraformaldehyde in PBS at 4 °C for 2 h. After fixation, tissues were incubated overnight at 4 °C in 30% sucrose, embedded in O.C.T. compound (Tissue-Tek), and frozen in a cryostat chamber at −35 °C. The samples were then cut into 6 µm thick frozen sections using a CM3000 cryostat (Leica, Wetzlar, Germany).

### 2.3. Measurement of the Relative Thickness of the Lamina Propria on Paraffin Sections of Urinary Bladder Tissue

To assess inflammation in the bladder wall based on the presence or extent of oedema, two H&E-stained sections of bladder tissue per animal (*n* = 3) from each group (Ctrl, CYP, CYP + veh, CYP + CBD) were photographed using a Nikon SMZ 800 stereomicroscope (Nikon, Amsterdam, The Netherlands). The images were analysed using the ImageJ programme (1.54d version; National Institutes of Health, Laboratory for Optical and Computational Instrumentation, University of Wisconsin, Madison, WI, USA). For each tissue section, the surface area (S1) of the lamina propria and the total surface area (S2) of the bladder wall (excluding the urothelium) were measured. The relative thickness of the lamina propria (rTh (LP)) in the bladder wall was then calculated using the following equation:

$$rTh\;(LP)\;[\%]=\frac{S1}{S2}\times 100$$

### 2.4. Immunofluorescence Labelling on Cryosections of Urinary Bladder Tissue

The frozen cryosections were first rinsed in PBS for 15 min. After permeabilisation with 0.4% Triton X-100 in PBS for 5 min at room temperature, the samples were washed in PBS (2 × 5 min) and incubated in blocking buffer (10% goat serum in PBS) for 1 h at 37 °C. Primary antibodies (Table 1), diluted in 1% BSA in PBS, were applied to the sections overnight at 4 °C. After rinsing in PBS (3 × 5 min), secondary antibodies (Table 1), diluted in 1% BSA in PBS, were applied and incubated for 1 h at 37 °C in the dark. The sections were washed again in PBS (3 × 5 min) and protected from light. Samples were mounted in Vectashield anti-bleaching mounting medium with DAPI (Vector Laboratories, Burlingame, CA, USA) for DNA staining and observed with an AxioImager Z1 fluorescence microscope (Carl Zeiss, Oberkochen, Germany).

### 2.5. Quantitative Analysis of Proliferative Activity of the Urothelium

To analyse proliferative activity of urothelial cells, two cryosections of bladder tissue per animal (*n* = 3) from each group (Ctrl, CYP, CYP + veh, CYP + CBD) were immunolabeled for Ki-67. To assess proliferative activity, urothelial cells with a positive immunohistochemical reaction for Ki-67, as well as all urothelial cells in each tissue section, were counted using a Nikon Eclipse E200 bright-field microscope (Nikon, Amsterdam, The Netherlands) or an AxioImager Z1 fluorescence microscope (Carl Zeiss, Oberkochen, Germany). From these values, the proliferative index (PI) was calculated using the following equation:

$$PI\;[\%]=\frac{Number\;of\;Ki67\text{-}positive\;urothelial\;cells}{Number\;of\;all\;urothelial\;cells\;per\;tissue\;section}\times 100$$

### 2.6. Quantitative Histopathological Assessment of Inflammatory Cells in the Bladder Wall

Paraffin blocks containing bladder samples from experimental animals of all four groups (*n* = 4 for Ctrl, CYP + veh, and CYP + CBD groups; *n* = 6 for CYP group) were cut into 4 μm-thick sections using a microtome, deparaffinised, and stained with hematoxylin and eosin according to the standard protocol. The histological slides were digitised with an optical scanner (NanoZoomer S360, Hamamatsu Photonics K.K., Hamamatsu, Japan). Two consecutive sections of the urinary bladder from each animal were examined with a light microscope (Eclipse E600, Nikon, Tokyo, Japan) at 400× magnification. In parallel, the digitised slides were analysed on a computer using NanoZoomer S360 viewing software (NDP serve 3.3.38) at 400× magnification.

To ensure unbiased histopathological evaluation, the samples and histological slides were coded until the evaluation of all bladder specimens was completed. For each sample, the total area of the lamina propria (i.e., excluding the urothelium and muscular layer of the bladder wall) was measured using NDP.view2 software (version 3.3.26, Hamamatsu Photonics K.K., Hamamatsu, Japan), and the result was presented in mm^2^. Extravascular inflammatory cells (lymphocytes, monocytes, and neutrophils) were counted throughout the entire lamina propria of the bladder, and the results were expressed as the number of inflammatory cells per mm^2^. All measurements were performed by the same pathologist, who was blinded to the treatment of the animals.

### 2.7. Sample Preparation for Scanning Electron Microscopy (SEM)

Bladder tissue pieces were fixed in a solution of 2% paraformaldehyde and 2% glutaraldehyde in 0.2 M cacodylate buffer for 3 h at 4 °C, then post-fixed in a 1:1 mixture of 2% OsO_4_ and 0.2 M cacodylate buffer for 1 h in the dark at room temperature. Samples were dehydrated through a graded acetone series, dried using CO_2_ at the critical point, sputter-coated with gold, and mounted on aluminium holders with silver paste for imaging with a Tescan Vega3 scanning electron microscope (Tescan, Brno, Czech Republic) at 30 kV.

### 2.8. Sample Preparation for Transmission Electron Microscopy (TEM)

Bladder tissue pieces (1 mm^3^) were prepared for ultrastructural analysis by fixation in 4.5% paraformaldehyde and 2% glutaraldehyde in 0.2 M cacodylate buffer (pH 7.3) for 3 h at 4 °C, followed by post-fixation with 1% OsO_4_ in 0.2 M cacodylate buffer for 1 h in the dark at room temperature and staining with uranyl acetate. Samples were dehydrated through an ethanol series, infiltrated with propylene oxide and Epon resin (Serva, Electrophoresis, Heidelberg, Germany), and polymerised at gradually increasing temperatures. Semi-thin sections were first cut and stained with toluidine blue for light microscopy to identify regions of interest. Ultrathin sections (50–60 nm) were then cut, mounted on grids, and contrasted with uranyl acetate and lead citrate for examination with a Philips CM100 transmission electron microscope (Philips, Eindhoven, The Netherlands) at 80 kV.

### 2.9. RNA Isolation and Quantitative PCR Analysis

Bladder halves obtained from 18 mice (Ctrl: *n* = 3; CYP: *n* = 3; CYP + veh: *n* = 6; CYP + CBD: *n* = 6) were frozen in liquid nitrogen and stored at −80 °C until further analysis. Tissue was homogenised in Qiazol lysis buffer with a 5 mm stainless steel bead in a Tissue Lyser LT (Qiagen, Hilden, Germany), and total RNA was isolated from the homogenate using the RNeasy Micro Kit (Qiagen, Hilden, Germany) according to the manufacturer’s instructions. The purity and quantity of RNA were determined using NanoDrop (Thermo Scientific, Waltham, MA, USA), measuring the OD ratio at 260 to 280 nm. Reverse transcription of 1 μg total RNA was performed using the First Strand cDNA Synthesis Kit (Roche, Mannheim, Germany) with oligo (dT) primers, and qPCR was conducted with a LightCycler 480 (Roche, Mannheim, Germany) in triplicate using 200 nM specific primers (Table 2), 10 ng cDNA, and EvaGreen qPCR Mix Plus (Solis, Estonia, Tartu, Estonia). Controls without a template and melting curves were measured with each plate. Data analysis was performed using the comparative ΔCt method with normalisation to the level of L32 [33].

### 2.10. Measurement of the Transepithelial Electrical Resistance

Transepithelial electrical resistance (TEER) of the urothelium was measured using bladder halves obtained from 4–8 mice per experimental group (Ctrl: *n* = 8; CYP: *n* = 4; CYP + veh: *n* = 4; CYP + CBD: *n* = 4). Immediately after excision, urinary bladders were placed in Dulbecco’s modified Eagle’s medium (DMEM; Sigma-Aldrich Chemie, Steinheim, Germany). Each bladder was bisected, and the halves were mounted on inserts with a circular aperture (2 mm diameter; surface area 0.031 cm^2^) and positioned between two EasyMount^®^ half-chambers of a diffusion chamber system (Physiologic Instruments, San Diego, CA, USA). All experiments were conducted at 36–37 °C. Incubation solutions were continuously oxygenated and mixed by bubbling with a gas mixture containing 95% O_2_ and 5% CO_2_.

**Scheme 2:**
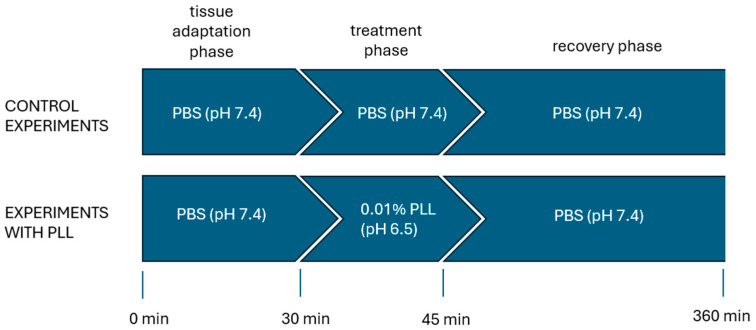
Schematic overview of the three experimental phases during which transepithelial electrical resistance of the urothelium was measured in a diffusion chamber system. The solutions to which the urothelial surface was exposed in each phase are indicated, while the serosal surface of the bladder tissue was continuously maintained in Dulbecco’s modified Eagle’s medium throughout the experiment. PBS, phosphate-buffered saline; PLL, poly-L-lysine.

Throughout the experiment (360 min), the serosal side of the bladder tissue was bathed in DMEM, which served as the acceptor solution. In control experiments, the urothelial surface was exposed to phosphate-buffered saline (PBS; pH 7.4) for the entire measurement. In treatment experiments, the urothelium was first exposed to PBS for 30 min to allow adaptation, followed by a 15 min treatment phase during which PBS was replaced with 0.01% poly-L-lysine in PBS (PLL; molecular weight 30–70 kDa, pH 6.5; Sigma-Aldrich Chemie, Steinheim, Germany). PLL was used to induce selective, concentration-dependent desquamation of superficial urothelial cells, as described in our previous study [34]. After treatment with PLL, during the recovery phase (315 min), the urothelium was again exposed to PBS (Scheme 2).

Electrophysiological parameters were recorded using a multichannel voltage–current clamp (VCC MC6, Physiologic Instruments, San Diego, CA, USA). Diffusion chambers were fitted with two pairs of Ag/AgCl electrodes connected via 3 M KCl/3.5% agar bridges for measurement of transepithelial potential difference and applied current. Electrical recordings were taken every 10 min during the adaptation phase, every 5 min during the treatment phase, and every 10 min during the first 120 min of the recovery phase. Subsequently, measurements were taken every 20 min until the experiment was completed at 360 min.

Electrical resistance was calculated according to Ohm’s law. Fluid resistance, determined before mounting the tissue in the diffusion chambers, was subtracted from the total measured resistance. The resulting net urothelial resistance was multiplied by the exposed tissue area (0.031 cm^2^) to obtain TEER values. To ensure comparability between samples, TEER measurements were normalised and expressed as a percentage of the TEER value recorded at the end of the equilibration phase for each tissue.

### 2.11. Assessment of Animal Burrowing Activity

The burrowing behaviour of animals from the CYP + veh (*n* = 9) and CYP + CBD (*n* = 8) groups was evaluated. The detailed protocol for measuring this spontaneous activity as an indicator of overall well-being is described in the Supplemental Material. Briefly, the animals’ burrowing activity was recorded every two days throughout the experiment. Animals were placed in individual cages containing a burrowing apparatus (a standard water bottle filled with pelleted diet). They were allowed to burrow for two hours every other day, for a total of two basal and five experimental measurements, starting with the first experimental measurement on the day of the first CBD or vehicle application (day 1; Scheme 1). Activity was assessed by weighing the remaining food inside the bottle at the end of each measurement and subtracting this from the weight of food before the start of the measurement. The amount of food burrowed at each experimental time point was normalised to the mean value of the two basal measurements (representing baseline burrowing activity), and the relative value was used for subsequent statistical analysis.

### 2.12. Statistical Analysis

Statistical analysis was performed using GraphPad Prism 8.0 and IBM SPSS Statistics 29.0. The Shapiro–Wilk test was used to evaluate the normality of data distribution. For group comparisons of proliferative activity, relative thickness of the lamina propria, and inflammatory cell infiltration, one-way ANOVA was used, followed by the Mann–Whitney test for within-group comparisons. Group differences in body mass data were analysed by one-way ANOVA, followed by the Holm–Sidak multiple comparisons test for post hoc pairwise comparisons. For burrowing activity, multiple *t*-tests were conducted to compare data at each time point between groups. To compare burrowing data within an individual group, the percentage of burrowed pellets at each time point was compared to the baseline value using repeated measures one-way ANOVA with Dunnett’s multiple comparisons test. For TEER data, Welch’s ANOVA was used to compare group means, with the Games–Howell multiple comparison test for post hoc analysis. For qPCR data (four to six measurements per group), Welch’s ANOVA was applied, followed by Dunnett’s T3 test for post hoc comparisons. A *p*-value < 0.05 was considered statistically significant.

## 3. Results

### 3.1. Effect of CBD on Damaged Urothelium

#### 3.1.1. Histological Analysis

**Figure 1:**
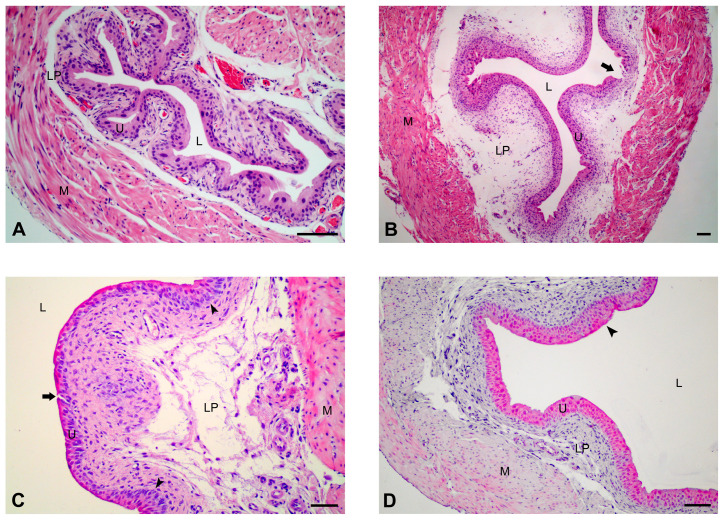
Representative histological micrographs showing the urothelium in urinary bladders from all experimental groups of mice. (**A**) In the bladder of a control mouse, the bladder lumen is lined by a three-layered urothelium. (**B**) Extensive and deep injury to the basal lamina is present (arrow) in the urothelium of a CYP-treated mouse. (**C**) The urothelium is hyperplastic (arrowheads) in some areas, with minor but deep injury (arrow), and clearly visible enlarged intercellular spaces between urothelial cells (arrowheads) in the bladder of a CYP + veh-treated mouse. (**D**) In the bladder of a CYP + CBD-treated mouse, the urothelium is mostly three-layered with occasional hyperplastic regions (arrowheads) and no injuries. L—lumen, U—urothelium, LP—lamina propria, M—muscle layer. H&E staining. Scale bars: (**A**,**C**,**D**) 50 µm; (**B**) 100 µm.

The urothelium of the Ctrl mice was three-layered, consisting of basal, intermediate, and large superficial cells (Figure 1A). In the CYP group, the urothelium was hyperplastic in some areas, with variably deep focal injuries (Figure 1B). In the CYP + veh group, the urothelium exhibited similar features to those in CYP-treated bladders, with hyperplastic and damaged regions (Figure 1C). In the CYP + CBD group, the urothelium was predominantly normoplastic, with occasional hyperplastic regions and no injuries (Figure 1D).

#### 3.1.2. Scanning Electron Microscopy (SEM) Analysis

**Figure 2:**
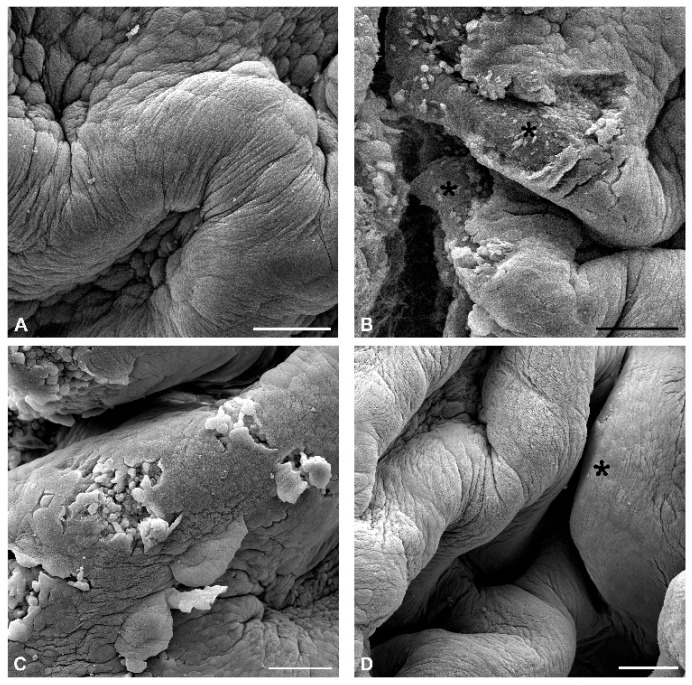
Representative SEM micrographs of the urothelium in urinary bladders from all experimental groups of mice. (**A**) The luminal surface of the urothelium in the control mouse consists of highly differentiated polygonal superficial cells with a typically scalloped apical plasma membrane and well-defined borders between them due to numerous tight junctions. (**B**) Extensive and deep injuries (asterisks) with urothelial cell desquamation extending to the basal lamina in the urothelium of the CYP-treated mouse. (**C**) Small and shallow injuries extending to the basal cell layer in the urothelium of the CYP + veh-treated mouse. (**D**) The luminal surface of the urothelium is intact, with an area of small and less differentiated superficial cells (asterisk) due to local renewal in the CYP + CBD-treated mouse. Scale bars: (**A**–**D**) 100 µm.

Morphological analysis of the urothelial surface by SEM revealed large polygonal, highly differentiated superficial cells, with well-developed tight junctions between them in the urinary bladders of Ctrl mice (Figure 2A). In mouse bladders treated with CYP, various injuries due to massive desquamation of the urothelial cells were detected, ranging from small and shallow to large and deep, with exposed basal lamina (Figure 2B). In the bladders of the CYP + veh group, the urothelial surface was predominantly intact, with occasional local and shallow injuries (Figure 2C). In the urinary bladders of the CYP + CBD group, the urothelial surface was intact, with some areas of renewal after injury, where smaller and less differentiated superficial cells were present on the surface (Figure 2D).

#### 3.1.3. Transmission Electron Microscopy (TEM) Analysis

**Figure 3:**
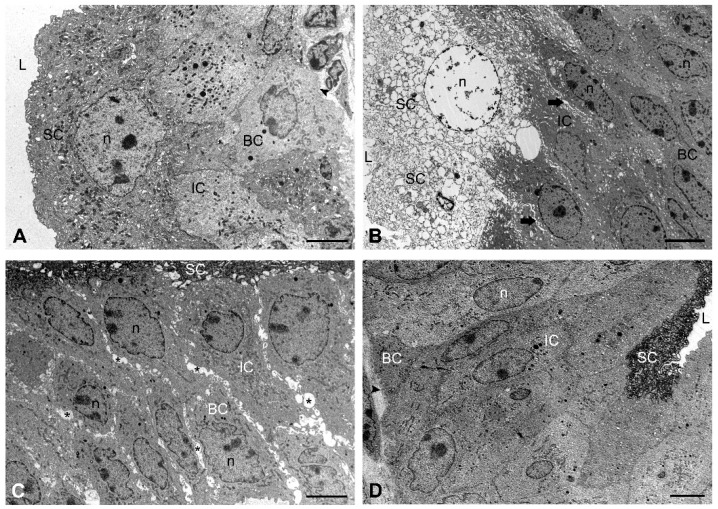
Representative TEM micrographs of the urothelium in urinary bladders from all experimental groups of mice. (**A**) The three-layered urothelium of the control mouse consists of undifferentiated basal cells at the basal lamina, intermediate cells, and large superficial cells lining the bladder lumen. A high stage of superficial cell differentiation is indicated by numerous specific fusiform vesicles in the cytosol and a typically scalloped apical plasma membrane. (**B**) Necrotic superficial cells with extensive vacuolisation and chromatin disintegration are present at the injured area of the urothelium in the CYP-treated mouse. Note the enlargement of intercellular spaces between intermediate cells (arrows). (**C**) Marked enlargement of intercellular spaces between all urothelial cells (asterisks) is observed due to disassembled intercellular junctions in the urothelium of the CYP + veh-treated mouse. (**D**) The architecture and ultrastructure of the urothelium in the CYP + CBD-treated mouse are normal and resemble those in control mice. Arrowhead—basal lamina. L—lumen of the bladder, SC—superficial cell, IC—intermediate cell, BC—basal cell, n—nucleus. Scale bars: (**A**–**D**) 6 µm.

TEM analysis of the urothelium in control mice confirmed the typical ultrastructural characteristics of all three types of urothelial cells and well-developed intercellular junctions between them (Figure 3A). In the urothelium of CYP-treated mice, numerous necrotic or viable desquamated cells were observed, with disrupted cell junctions and enlarged intercellular spaces in the injured area (Figure 3B). In the bladders of the CYP + veh mouse group, the urothelium with complete architecture was mostly normoplastic or hyperplastic in some areas, but with enlarged intercellular spaces in all cell layers due to interrupted cell junctions (Figure 3C). The urothelium of CYP + CBD-treated mice was mostly three-layered, with some hyperplastic regions of four to five cell layers and with functional intercellular junctions between the cells, including tight junctions between superficial cells. The population of superficial cells was heterogeneous and comprised large, highly differentiated cells in normoplastic urothelial regions and smaller, partially differentiated cells in hyperplastic areas of the urothelium (Figure 3D).

#### 3.1.4. Analysis of Proliferative Activity

**Figure 4:**
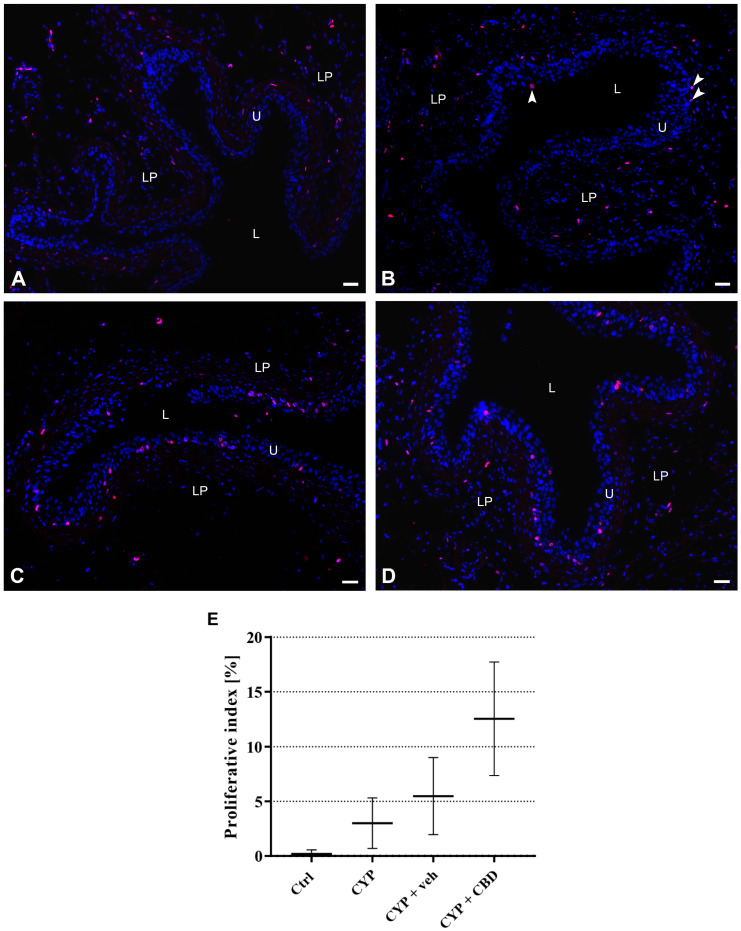
Proliferative activity of urothelial cells in the urinary bladders from all experimental groups of mice. (**A**–**D**) Representative micrographs of immunofluorescence labelling of Ki-67 (red fluorescence) and nuclear staining with DAPI (blue fluorescence). (**A**) No nuclei of urothelial cells with positive Ki-67 immunoreaction (pink colour due to merged red and blue fluorescence) are observed in the urothelium of the control group. (**B**) Only the nucleus of a superficial cell (separate arrowhead) and two basal cells (aligned arrowheads) with positive Ki-67 immunoreaction (pink colour due to merged red and blue fluorescence) are present in the urothelium of CYP-treated mice. (**C**) Numerous nuclei of urothelial cells with positive Ki-67 immunoreaction (pink colour due to merged red and blue fluorescence) are seen in the basal and intermediate cell layers of the urothelium of CYP + veh-treated mice. (**D**) Numerous nuclei of urothelial cells with positive Ki-67 immunoreaction (pink colour due to merged red and blue fluorescence) are present in all layers of the urothelium of CYP + CBD-treated mice. L—lumen of the bladder, U—urothelium, LP—lamina propria. Scale bars: (**A**–**D**) 50 µm. (**E**) Chart of proliferative indexes (PI) for each animal group (*n* = 3 per group). Data are presented as mean values and standard deviations for each experimental group of mice.

The results of immunolabeling for the proliferative marker Ki-67 and subsequent quantitative analysis of the proliferative activity of urothelial cells (Ki-67-positive) confirmed the well-known quiescent nature of the urothelium in Ctrl mice, as no Ki-67-positive cells were present in the urothelium (Figure 4A) and the mean proliferative index (PI) was close to zero (PI = 0.28) (Figure 4E). Proliferative activity of urothelial cells in CYP-treated mouse bladders was slightly higher (PI = 2.99) (Figure 4E), and only rare Ki-67-positive cells were detected in the urothelium (Figure 4B). In the bladders of CYP + veh mice, numerous Ki-67-positive urothelial cells were found in the basal and intermediate cell layers (Figure 4C), and the mean PI value was 5.48 (Figure 4E). The most intense proliferative activity of urothelial cells was observed in the bladders of CYP + CBD mice, where the mean PI was 12.54 (Figure 4E) and Ki-67-positive cells were found in all cell layers of the urothelium (Figure 4D).

### 3.2. Effect of CBD on Compromised Barrier Function of the Urothelium

**Figure 5:**
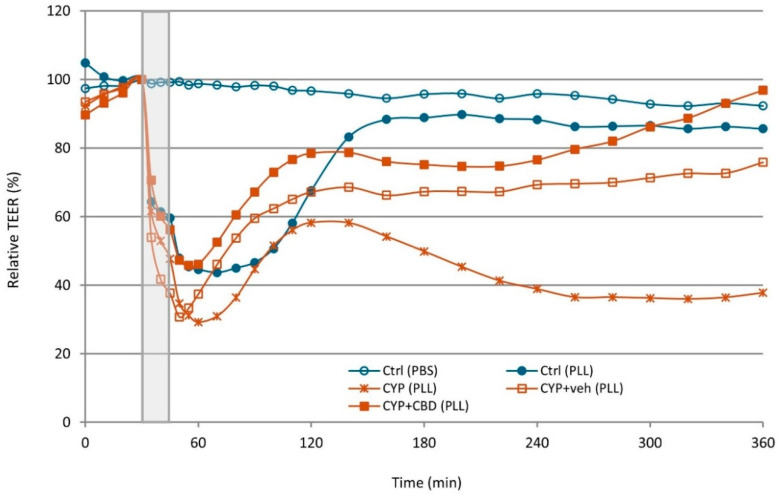
Changes in relative transepithelial electrical resistance (TEER) of the urothelium during the 6 h experimental period. Ctrl, control group of mice; CYP, cyclophosphamide-treated group of mice; CYP + veh, cyclophosphamide and vehicle-treated group of mice; CYP + CBD, cyclophosphamide and CBD-treated group of mice. The grey shaded area indicates the 15 min treatment phase, during which the apical surface of the urothelium was exposed to either phosphate-buffered saline (PBS) or poly-L-lysine (PLL). One group of Ctrl bladders (PBS) served as a positive control without PLL treatment, demonstrating the viability of the bladder urothelium throughout the ex vivo experiment. Data are presented as mean relative TEER values for each group (*n* = 4 per group).

TEER was measured ex vivo over 6 h (Figure 5). A fifteen-minute exposure of the urothelium to PLL caused a marked reduction in relative TEER values across all experimental groups. This decrease was more pronounced in the CYP group than in the Ctrl group. In CYP-exposed animals, treatment with CBD (CYP + CBD group) attenuated the PLL-induced reduction in TEER, whereas treatment with vehicle alone (CYP + veh group) did not prevent the decline of TEER values. However, no statistically significant differences were observed among PLL-exposed groups at 45 and 60 min (Table 3).

During the regeneration phase, the bladders of the Ctrl group showed the fastest recovery towards baseline TEER values among PLL-exposed groups. Efficient TEER restoration was also observed in both the CBD and vehicle bladders; however, the bladders of the CYP + CBD group consistently achieved higher mean TEER values than those of the CYP + veh group. In contrast, TEER values of the bladders of the CYP group remained significantly reduced throughout the regeneration phase and continued to decline over time. A statistically significant difference in relative TEER values among PLL-exposed bladders was observed at 180 min, whereas this difference was no longer significant at the end of the experiment (360 min), mainly due to the small sample size and high variability (standard deviation) of the measurements (Table 3).

### 3.3. Effect of CBD on Chronic Bladder Inflammation

#### 3.3.1. Quantitative Assessment of Oedema in the Urinary Bladder Wall

**Figure 6:**
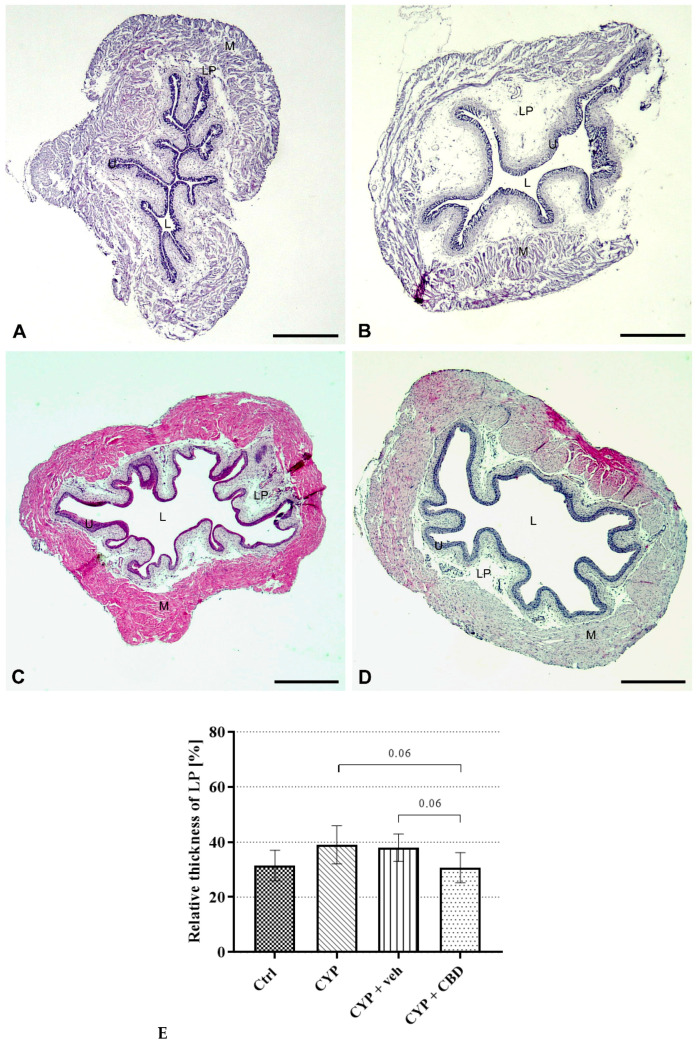
Representative micrographs taken with a stereomicroscope showing the thickness of the lamina propria in urinary bladders from all experimental groups of mice. In the bladder wall of a control mouse, the lamina propria is thin (**A**), while in the bladder of a CYP-treated mouse, the lamina propria is thickened due to prominent oedema (**B**). In the bladder of a CYP + veh-treated mouse, most of the lamina propria is swollen due to moderate oedema (**C**), whereas in the bladder of a CYP + CBD-treated mouse, only mild oedema is present in some areas, while in others the lamina propria is as thin as in control mice (**D**). L—lumen, U—urothelium, LP—lamina propria, M—muscle layer. H&E staining. Scale bars: (**A**–**D**) 1 mm. (**E**) Differences in measured relative areas of the lamina propria in the urinary bladders of the Ctrl (*n* = 3), CYP (*n* = 3), CYP + veh (*n* = 3), and CYP + CBD (*n* = 3) groups of mice. Data are presented as mean values and standard deviations for each experimental group.

The results of histomorphological analysis of bladder wall thickening due to oedema of the lamina propria, the most prominent sign of inflammation, revealed the greatest oedema in bladders of CYP-treated mice, less extensive oedema in bladders of CYP + veh-treated mice, and almost no oedema in CYP + CBD-treated mice (Figure 6A–D).

The quantitative results obtained by measuring the relative area of the lamina propria in the bladder wall confirmed our histomorphological findings. The differences indicated a trend towards oedema reduction in CYP + CBD-treated mouse bladders compared to CYP and CYP + veh-treated mice. However, the measured relative thickness of the lamina propria in CYP + CBD mice (mean value 30.68%) was comparable to that in control mice (mean value 31.45%) (Figure 6E).

#### 3.3.2. Histopathological Analysis of Extravascular Inflammatory Cells in the Lamina Propria

**Figure 7:**
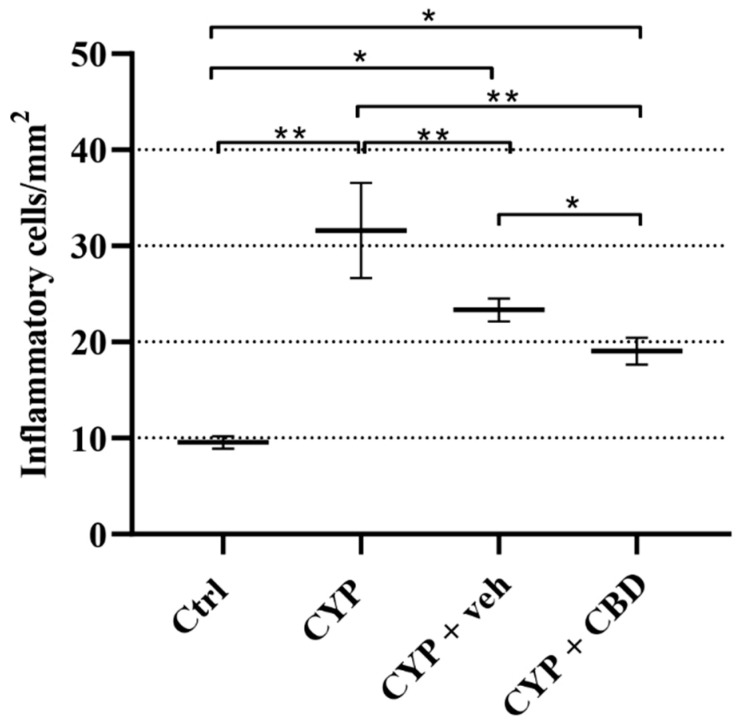
Number of infiltrated inflammatory cells in the lamina propria of Ctrl (*n* = 4), CYP (*n* = 6), CYP + veh (*n* = 4), and CYP + CBD (*n* = 4) groups of mice. The number of inflammatory cells is normalised per mm^2^. Data are presented as mean values and standard deviations for each experimental group of mice. * *p* < 0.05; ** *p* < 0.01.

In the control group, scattered extravascular inflammatory cells (lymphocytes and monocytes) were observed, with no neutrophils present. In the experimental groups, infiltrated lymphocytes and monocytes predominated, while neutrophils were few and mast cells were scarce. The number of infiltrated inflammatory cells differed significantly between the groups. The mean number of inflammatory cells detected in the lamina propria of control mice was 9.55 cells/mm^2^. The highest number of inflammatory cells was found in the lamina propria of CYP-treated mice (31.60 cells/mm^2^). Compared to those, the inflammatory cell infiltration was significantly lower in the CYP + veh and CYP + CBD groups, with mean values of 19.05 and 23.35 cells/mm^2^, respectively. However, the number of infiltrated inflammatory cells was significantly lower in the lamina propria of CYP + CBD animals compared to CYP + veh-treated animals (Figure 7).

#### 3.3.3. Expression of Cannabinoid CB1 and CB2 Receptors in the Bladder Wall

(a) Analysis of CB1R and CB2R mRNA expression

**Figure 8:**
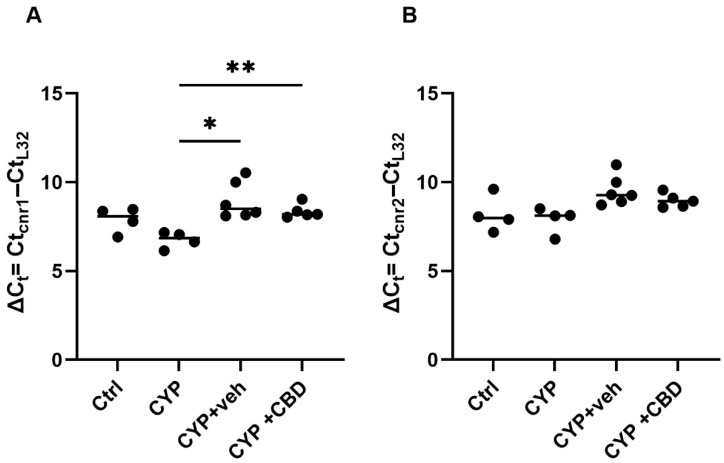
The mRNA expression of CB1 receptor (**A**) and CB2 receptor (**B**) in mouse urinary bladders of each group (Ctrl: *n* = 4 per group, CYP: *n* = 4 per group, CYP + veh: *n* = 6 per group, and CYP + CBD: *n* = 5 per group) analysed by qPCR and normalised to the expression of L32 using the comparative method; * *p* < 0.05; ** *p* < 0.01.

While the mRNA expression of CB1R was significantly deregulated in response to various treatments of mice (Figure 8A), the mRNA expression of CB2R remained stable regardless of animal treatment (Figure 8B). Specifically, the mRNA level of CB1R decreased after CYP treatment, while vehicle or CBD treatment led to a statistically significant increase in CB1R mRNA levels compared to the CYP group (*p* = 0.0134 and *p* = 0.0078, respectively), reaching levels similar to those of the control group.

(b) Analysis of CB1R and CB2R protein expression

**Figure 9:**
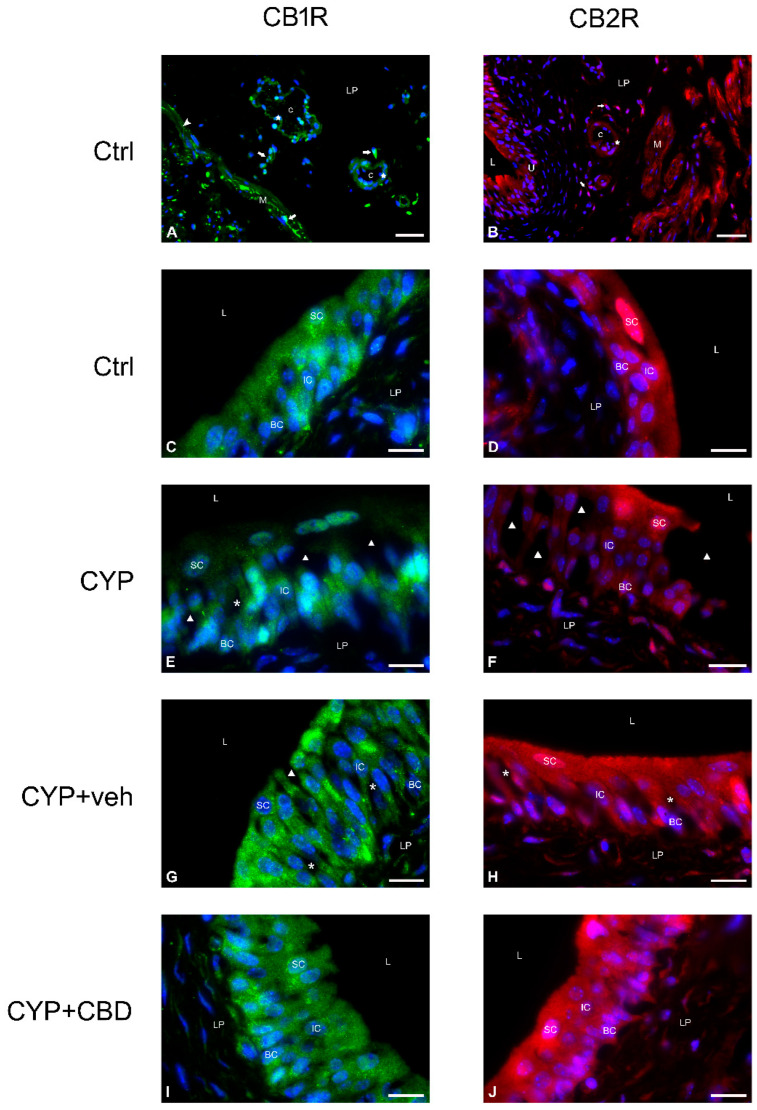
Representative micrographs of immunofluorescence labelling of CB1 and CB2 cannabinoid receptors in the bladder wall (**A**,**B**) and in the urothelium (**C**–**J**) of all experimental groups of mice. (**A**,**C**,**E**,**G**,**I**) Positive immunofluorescence reaction for CB1R is shown in green, and nuclei are shown in blue due to DAPI staining. (**B**,**D**,**F**,**H**,**J**) Positive immunofluorescence reaction for CB2R is shown in red, and nuclei are shown in blue due to DAPI staining. L—lumen of the bladder, U—urothelium, SC—superficial cell, IC—intermediate cell, BC—basal cell, LP—lamina propria, M—muscle cell, arrow—interstitial cell, triangle—urothelial injury, asterisk—dilated intercellular space, star—endothelial cell, arrowhead—nerve ending, c—capillary. Scale bars: (**A**,**B**) 50 µm; (**C**–**J**) 20 µm.

Immunofluorescence labelling of cannabinoid CB1 and CB2 receptors in urinary bladder tissues from control, CYP + veh, and CYP + CBD groups of mice revealed strong expression of both receptor types in all urothelial cells, while weaker expression of both receptors was observed in some urothelial cell layers of CYP-treated mice. The immunoreactions for both receptors were detected mainly in the cytoplasm of all urothelial cells. However, positive immunoreactions for CB1R in the urothelium of CYP-treated mice and for CB2R in the urothelium of all experimental mice were also detected in the nuclei of superficial urothelial cells (Figure 9, Table 4 and Table 5).

In other structures of the bladder wall, the immunoreactions for both types of cannabinoid receptors differed in presence and intensity. Specifically, CB1R-positive immunoreaction was observed in interstitial cells of the lamina propria, endothelial cells, and nerve fibres, whereas muscle cells of the detrusor were CB1R-negative in the bladders of all four groups of mice (Figure 9, Table 4). CB2R-positive immunoreaction was found in interstitial cells of the lamina propria, endothelial cells, and muscle cells, but nerve fibres were CB2R-negative in the bladders of all four groups of mice. Less intense anti-CB1R and anti-CB2R immunoreactions were observed in some suburothelial structures only in CYP-treated mice (Figure 9, Table 5).

### 3.4. Effect of CBD on Bladder-Related Pain in Experimental Animals

#### 3.4.1. Animal Body Weight Monitoring

Monitoring body weight provides a reliable and objective measure of animal well-being throughout the experiment. To assess the impact of CBD on animal health, the percentage of body weight maintained at the end of the experiment was calculated relative to the body weight at the beginning of the experiment for each animal.

**Figure 10:**
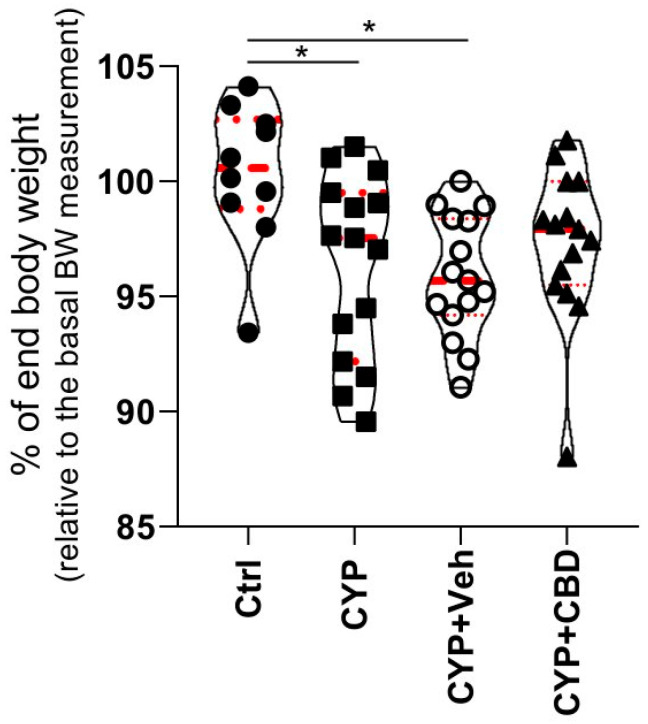
Comparison of changes in animal body weight. The mean percentage of maintained body weight is compared among animal groups (*n* = 10 for the Ctrl group; *n* = 15 for the CYP-treated group; *n* = 15 for the CYP + CBD group; *n* = 15 for the CYP + veh group). Violin plots show the percentage of final body weight for individual animals in each group. Red spotted lines show Q1, mean, and Q3. * *p* < 0.05.

The change in remaining body weight differed significantly between the mouse experimental groups (*p* = 0.0114). Post hoc analysis showed no significant difference for the CYP + CBD group (*p* = 0.1285; mean percentage of maintained weight 97.30; Figure 10) compared with the Ctrl group (mean percentage of maintained weight 100.3). In contrast, the change in body weight was significant for the CYP group (*p* = 0.0239; mean percentage of maintained weight 96.33) and the CYP + veh group (*p* = 0.0102; mean percentage of maintained weight 95.90) compared with the Ctrl group.

#### 3.4.2. Analysis of Animal Burrowing Activity

The impact of CBD on bladder pain in experimental mice with CYP-induced cystitis was indirectly assessed by quantitatively measuring of burrowing activity. Mice treated with either CBD or vehicle were exposed to the burrowing setup every other day throughout the experiment.

**Figure 11:**
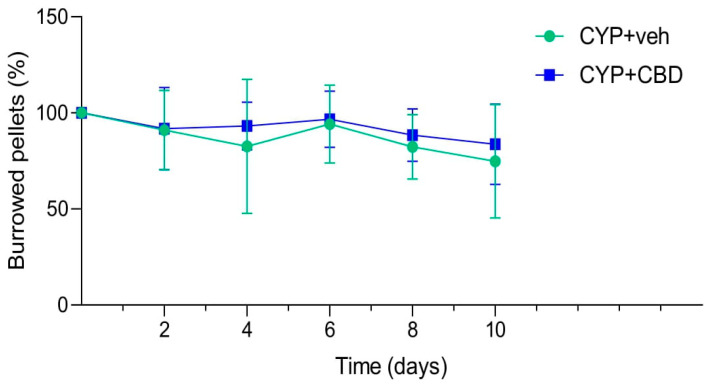
Summary statistics of measured burrowing activity in the CYP + veh group and CYP + CBD groups of mice during the experiment. Data at each time point are presented relative to basal burrowing activity, which is set at 100% for graphical presentation.

Burrowing activity did not differ significantly between CYP + CBD-treated mice and CYP + veh-treated mice at any time point during the experiment. However, mean burrowing activity was consistently higher in the CYP + CBD group than in the CYP + veh group at all time points (Table 6, Figure 11). Burrowing activity progressively decreased in both groups over the course of the experiment, reducing to a mean of 83.7% and 74.79% for the CBD and vehicle groups, respectively. However, burrowing activity of CYP + CBD-treated mice was not significantly reduced at any time point compared to the baseline value (set at 100%), while the reduction was significant only on day 8 for the CYP + veh group (*p*_adj_ = 0.0489; Table 6).

## 4. Discussion

IC is characterised by bladder inflammation, frequent urination, and chronic pelvic pain and pressure. CBD, a non-psychoactive cannabinoid, is known to have anti-inflammatory, antioxidant, and immunomodulatory effects, demonstrated in a wide range of pathological conditions, including various neuropsychiatric disorders, cardiovascular disease, and cancer [35,36,37,38]. Therefore, we hypothesised that CBD could have beneficial effects in alleviating signs of bladder inflammation, which remains an understudied area of cannabinoid research. In this study, a well-established model of experimentally induced aseptic chronic cystitis [32] was used, employing a bladder-centric approach with repeated intraperitoneal administration of low-dose cyclophosphamide (CYP) combined with daily intraperitoneal administration of CBD. The main purpose of this study was to assess the effects of CBD on the urinary bladder, focusing on damaged urothelium and pronounced inflammation, as these are two typical signs of IC.

The primary goal of this study was to assess how CBD influences the regeneration of damaged urothelium, a common feature in patients with IC. Our comprehensive microscopic analysis at both histological and ultrastructural levels demonstrated that CBD significantly promoted the regeneration of CYP-induced damaged urothelium, likely by stimulating urothelial cell proliferation. Specifically, in the bladders of CBD-treated mice, no urothelial injury was observed, in contrast to vehicle-treated mice; the urothelium was hyperplastic in some areas and covered by numerous small, less differentiated superficial cells, indicating active renewal. Our evaluation of urothelial proliferative activity supported the morphological findings, revealing a trend towards increased urothelial cell proliferation in CBD-treated mice compared to other groups. Recently, Piao and colleagues reported a similar finding regarding CBD-induced suppression of urothelial injury at the histological level [31].

The second aim of our study was to assess the effect of CBD on impaired function of the urothelium as a blood–urine permeability barrier in IC. Barrier dysfunction was mimicked ex vivo by exposing bladders from all four animal groups to PLL and measuring TEER. Based on our previous studies, PLL induces selective and concentration-dependent desquamation of superficial urothelial cells, resulting in injuries similar to those observed in bladders in vivo [34]. Measurement of transepithelial permeability first revealed a pronounced reduction in relative TEER values of all bladders exposed to PLL. During the subsequent recovery phase, after replacing PLL with PBS, TEER values of bladders from both CBD-treated and vehicle-treated mice showed rapid and efficient restoration of the permeability barrier, reaching levels comparable to those observed in control bladders. However, bladders of the CYP + CBD group consistently achieved higher mean TEER values than those of the CYP + veh group. Therefore, it can be confirmed that CBD induces rapid restoration of the compromised blood–urine barrier, a finding consistent with those of Piao and colleagues [31].

The third objective of the study was to assess the potential influence of CBD on reducing oedema of the lamina propria, a typical tissue response to injury and a sign of inflammation, which occurs due to increased capillary permeability and fluid leakage into the surrounding tissue. Quantitative histological analysis of bladder wall thickening showed a marked reduction to only mild oedema in the bladders of CBD-treated mice. Thus, the trend towards oedema reduction in CBD-treated mice compared to CYP-treated and vehicle-treated mice indicates a specific anti-inflammatory effect of CBD. Additionally, we assessed the influence of CBD on bladder inflammation using quantitative histopathological analysis of inflammatory cells recruited from blood vessels in the lamina propria due to chemical mediators released by immune defence cells. The results of this analysis support our oedema measurements, as infiltration of inflammatory cells in the lamina propria of the CYP + CBD group was the lowest of all experimental groups and significantly lower compared to the CYP and CYP + veh groups, again demonstrating the beneficial anti-inflammatory effect of CBD. Our findings on the effect of CBD on the extent of bladder inflammation are consistent with studies reporting beneficial anti-inflammatory effects of CBD on bladder tissue and inflammatory pain [29,31]. Both research groups reported an association of CBD activity with inhibition of several mediators of inflammation and oxidative stress, such as prostaglandin E2, NO, TNFα, and COX-2, the latter also confirmed in in vitro models [30,39].

Studies suggest that the molecular mechanism of CBD action involves the endocannabinoid system, which includes two primary cannabinoid receptors, CB1R and CB2R. CB1R is mainly found in the nervous system, but is also present in adipocytes, pancreas, liver, and skeletal muscle, while CB2R is associated with anti-inflammatory and immunomodulatory effects and is found primarily in the immune system and spleen [40,41,42,43]. To evaluate the effects of inflammation induction and subsequent CBD treatment, the expression of CB1R and CB2R was examined at both gene and protein levels in all four experimental groups. We observed that CB1R mRNA expression varied significantly between some groups but not between the CYP + veh and CYP + CBD groups. mRNA levels were lower in CYP-treated mice compared to control mice, which contrasts with data from patients with chronic bladder pain syndrome [44]. However, CBD treatment led to a significant increase in CB1R mRNA levels compared to inflamed CYP-treated bladders. Conversely, our qPCR analysis revealed that CB2R mRNA expression in mouse bladders did not differ significantly among the experimental groups and was similar to that in control bladders, regardless of inflammation or CBD treatment. We can thus conclude that CBD treatment primarily influences the cannabinoid CB1 receptor by inducing CB1R mRNA expression. The decrease in CB1R mRNA expression and unchanged CB2R mRNA expression in bladders with CYP-induced inflammation observed in our study contrasts with the findings of Tambaro and colleagues [26], where LPS-induced bladder inflammation in mice increased mRNA expression of CB2 but not CB1 receptor. However, these discrepancies could be model-specific, as a variety of CB1R and CB2R responses to induced bladder inflammation have been reported [45].

To gain further insight into CB1R and CB2R protein expression in the bladder wall and to assess the effects of different treatments on their distribution, we performed immunolabeling of both receptor types. We found that CB1R and CB2R are localised in all urothelial cells, interstitial cells of the lamina propria, and endothelial cells of control bladders. However, their presence varied in specific cell types: CB2R was detected in muscle cells, while CB1R was present in nerve cells. Moreover, the distribution of neither CB1R nor CB2R changed with treatment and remained the same as in control urinary bladders, except in CYP-treated mice, where weaker expression of CB1R and CB2R was observed due to acute tissue injury in the bladder wall. In light of these observations, we conclude that CBD has no effect on CB2R gene expression or protein localisation in mouse bladders, while it increases the level of CB1R gene expression without altering its distribution within the mouse bladder wall. However, our results are not consistent with other data in the literature. There are many reports on the distribution and activation of cannabinoid CB1 and CB2 receptors by various ligands in the urinary bladder of animal models with experimental cystitis [14,15,16,17,25,26,27,28,46]. The results of our study and some others coincide mainly in the localisation of CB1 and CB2 receptors in the urothelium [16]. We consider that the reasons for discrepancies lie in the use of different animal types, different methods of cystitis induction, or variations in experimental setup, which make these data almost incomparable.

Finally, we aimed to assess the effect of CBD on the well-being of experimental animals by monitoring body weight and burrowing activity. Both methods are simple, non-invasive, and sensitive for detecting disturbances in rodent health and behaviour. The first test showed a drop in body weight in all treated mice, which was expected due to multiple daily interventions during the experiment. However, a statistically significant decrease was observed in the CYP-treated and vehicle-treated mice compared to control mice, but not in the CBD-treated mice, which may indicate a slight beneficial effect of CBD on the overall well-being of the animals. To address the second aspect of CBD’s effect on mouse well-being, we monitored burrowing activity, a natural behaviour of healthy rodents [47,48,49]. Our results show that, although burrowing activity did not differ significantly between vehicle-treated and CBD-treated mice, the mean burrowing activity was consistently higher in the CYP + CBD group than in the CYP + veh group at all time points during the experiments. We speculate that including a larger number of animals in the study would be necessary to achieve statistical significance between these two groups. However, due to the consistently higher activity of CBD-treated mice throughout the experiment, we assume that CBD has a beneficial effect on the general well-being of the animals, probably by reducing bladder pain.

The main limitation of our study is the small sample size for some methods, so our findings indicate only trends of increased proliferative activity and reduced oedema due to CBD. Larger animal studies are therefore required to confirm these results. Nonetheless, the study provides new insights into the positive effects of CBD on chronic urinary bladder inflammation in a mouse model of IC. To investigate this, we used a range of structural and functional methods at the molecular, tissue, and organismal levels. To our knowledge, this is the most comprehensive morphological analysis of the impact of CBD on damaged urothelial structure, one of the most prominent symptoms of IC in patients. It also presents the first report on the effect of CBD on urothelial function via measurement of urothelial permeability, laying the groundwork for future physiological studies of bladder function. As CBD administration began one day before the first CYP injection, our study design includes a minor prophylactic component. However, as most CBD treatment continued during cystitis induction (repeated injury and inflammatory insult), the observed effects of CBD clearly reflect modulation of the disease process and alleviation of key symptoms. Finally, the CBD dose used in our mouse model is within the safe and effective range for adults [50,51], which may help facilitate potential translation to patients with IC.

In summary, CBD promotes the restoration of urothelial structure and its compromised barrier function and alleviates chronic bladder inflammation. These findings highlight the promising therapeutic benefits of CBD, although further mechanistic and animal studies are required before safe translation into clinical practice.

## Figures and Tables

**Table 1:** List of primary and secondary antibodies with their dilutions and manufacturers.

Primary Antibodies	Secondary Antibodies
Anti-Ki-67 antigen (1:100; rabbit monoclonal; Abcam, Cambridge, UK; #ab16667)	Goat anti-rabbit (1:400; Alexa Fluor^®^ 555; Invitrogen, Carlsbad, CA, USA; #A21428)
Anti-CB1 receptor (1:100; rabbit polyclonal; Cayman Chemical; Ann Arbor; MI, USA; #10006590)	Goat anti-rabbit (1:400; Alexa Fluor^®^ 488; Invitrogen, Carlsbad, CA, USA; #A11008)
Anti-CB2 receptor (1:100; rabbit polyclonal; Cayman Chemical; Ann Arbor, MI, USA; #101550)	Goat anti-rabbit (1:400; Alexa Fluor^®^ 555; Invitrogen, Carlsbad, CA, USA; #A21428)

**Table 2:** Primers used in qPCR analysis.

Gene	Forward Primer	Reverse Primer
L32	CCTCTGGTGAAGCCCAAGATC	TCTGGGTTTCCGCCAGTTT
CBR1	AAGTCGATCTTAGACGGCCTT	TCCTAATTTGGATGCCATGTCTC
CBR2	ATGGCCGTGCTCTATATTATCCT	ATGGTCACACTGCCGATCTTC

**Table 3:** Mean values ± standard deviation of relative TEER (%) measured at specified time points for all animal groups exposed to poly-L-lysine during the treatment period. Results of one-way ANOVA at each time point are presented, with statistically significant *p*-values (*p* < 0.05) shown in bold. Post hoc analysis was performed to evaluate pairwise differences and revealed a statistically significant difference only between the two groups highlighted in red.

	Mean Values ± Standard Deviations				ANOVA
Time (min)	Ctrl (PLL)	CYP (PLL)	CYP + CBD (PLL)	CYP + veh (PLL)	*p*-Value
45	60 ±18	48 ± 17	56 ± 19	38 ± 16	0.295
60	45 ± 7	29 ± 15	46 ± 13	37 ± 17	0.313
120	68 ± 18	58 ± 15	79 ± 6	67 ± 21	0.383
180	89 ± 7	50 ± 15	75 ± 11	67 ± 28	**0.047**
360	86 ± 24	38 ± 16	97 ± 29	76 ± 41	0.063

**Table 4:** Qualitative results of immunodetection of CB1 receptors in the urinary bladders of mice from all experimental groups.

Mouse Experimental Groups	Urothelium			Suburothelial Structures of the Bladder Wall			
	SC	IC	BC	Interstitial Cells	Endothelial Cells	Muscle Cells	Nerve Fibres
Ctrl	++	++	++	++	+	-	++
CYP	+	++	+	++	+	-	+
CYP + veh	++	++	++	++	+	-	++
CYP + CBD	++	++	++	++	+	-	++

Legend: ++ strong immunofluorescence reaction against CB1 receptor; + weak immunofluorescence reaction against CB1 receptor; - negative immunofluorescence reaction against CB1 receptor; SC-superficial urothelial cells; IC-intermediate urothelial cells; BC-basal urothelial cells.

**Table 5:** Qualitative results of immunodetection of CB2 receptors in the urinary bladders of mice from all experimental groups.

Mouse Experimental Groups	Urothelium			Suburothelial Structures in the Bladder Wall			
	SC	IC	BC	Interstitial Cells	Endothelial Cells	Muscle Cells	Nerve Fibres
Ctrl	++	++	++	++	++	++	-
CYP	++	+	+	+	+	++	-
CYP + veh	++	++	++	++	++	++	-
CYP + CBD	++	++	++	++	++	++	-

Legend: ++ strong immunofluorescence reaction against CB1 receptor; + weak immunofluorescence reaction against CB1 receptor; - negative immunofluorescence reaction against CB1 receptor; SC-superficial urothelial cells; IC-intermediate urothelial cells; BC-basal urothelial cells.

**Table 6:** Mean values (%) of burrowing activity of the CYP + veh group of mice (*n* = 9) and the CYP + CBD group of mice (*n* = 8). The *p*-values at the right end of the table indicate differences between the CBD and vehicle groups at each time point. *p*-values at the bottom of the table indicate differences within each group when comparing mean burrowing activity at each time point to the baseline value. Day 8 of the experiment is the time point with a significantly different mean percentage of burrowing activity compared to the baseline activity shown in red (*p*_adj_ = 0.0489).

Time Point (Days)	CYP + veh Group Mean Value (%)	CYP + CBD Group Mean Value (%)	*p*-Value
2	91.06	91.82	0.942
4	82.60	93.15	0.432
6	94.16	96.70	0.774
8	82.37	88.48	0.428
10	74.79	83.70	0.489
***p*** ***-value***	0.2082	0.3488	

## Data Availability

The original contributions presented in this study are included in the article and Supplementary Material. Further inquiries can be directed to the corresponding author.

## Supplementary Materials

The following supporting information can be downloaded at: https://www.mdpi.com/article/10.3390/pharmaceutics18040458/s1, Supplementary Material S1: Animal burrowing: detailed protocol of the experiment.
