# Supplementary material for: CBD Promotes Structural and Functional Epithelial Restoration and Alleviates Inflammation in a Mouse Model of Interstitial Cystitis
Source: Pharmaceutics. 2026 Apr 9;18(4):458. doi: 10.3390/pharmaceutics18040458 (PMC13118637; doi:10.3390/pharmaceutics18040458)
Supplement: Supplementary file 1 [file pharmaceutics-18-00458-s001.zip › pharmaceutics-4202069-supplementary.pdf]

Supplementary material S1

Animal burrowing: detailed protocol of the experiment

Standard water bottles (250 mL) filled with 90 g of pelleted mouse diet were used as the burrowing apparatus. All exposures of the animals to the burrowing apparatus took place between 2 and 4 pm every 48 hours throughout the experiment. Burrowing activity was tested in the room where the animals were housed, without human presence.

The animals were first acclimatised to the burrowing apparatus as a novel object on day -7 (see Scheme 1 in the manuscript: Graphical presentation of the experimental design). Five pellet-filled bottles (the same number as the number of animals within a cage) were placed in the home cage and removed after 2 hours. After 48 hours (on day -5), each animal was individually exposed to the novel object in a separate cage. The purpose of this acclimation also served to familiarise the animals with short-term individual housing. To minimise stress, this acclimation lasted only 1 hour.

The cage setup is illustrated in the photos below. In addition to the burrowing apparatus, each cage was also equipped with paper towels and paper rolls for enrichment, a cover, and a water bottle. The same setup was used throughout the experiment, and each animal was assigned to the same cage for the duration of the experiment.

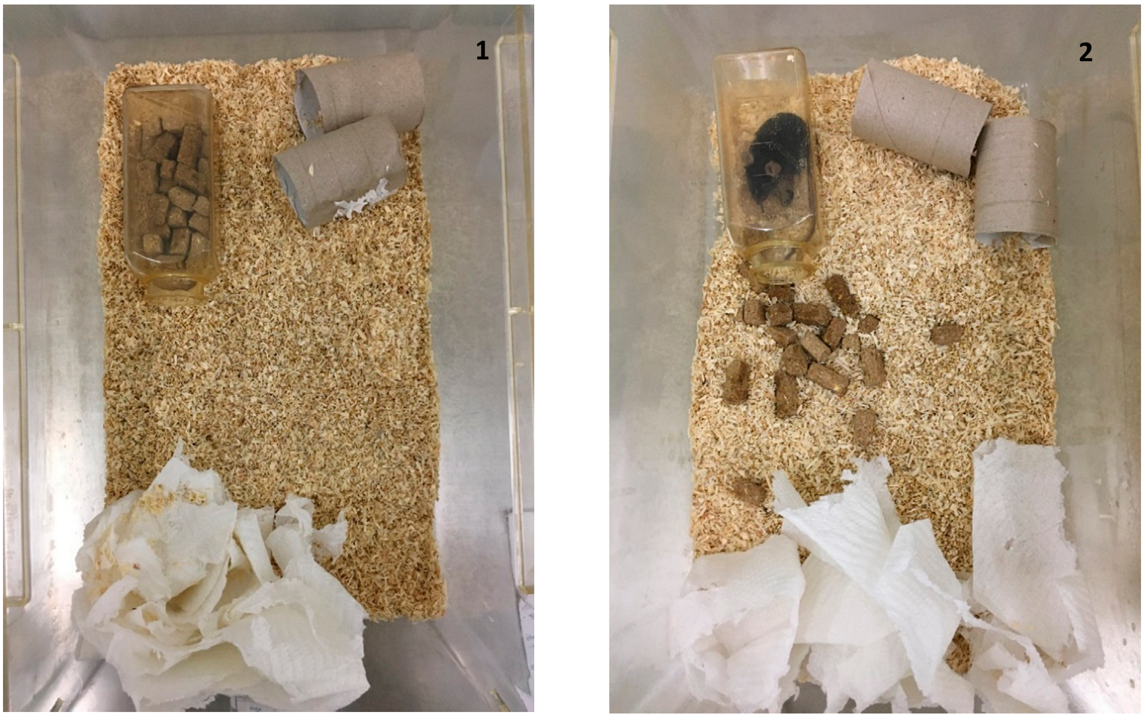

Supplementary Figure S1: Photos of 1) the experimental setup before the start of testing burrowing behavior, and 2) an example of a cage at the end of the burrowing test.

After acclimation, the burrowing activity of each animal was measured on two occasions before the first CBD administration (on days -3 and -1; see Scheme 1 in the manuscript: Graphical presentation of the experimental design) to establish baseline burrowing activity, followed by five experimental

measurements (on days 1, 3, 5, 7, and 9). First, the pellets in the burrowing apparatus were weighed. The animals were then placed in individual cages and allowed to burrow for two hours. At the end of the experiment, the animals were returned to their home cages. The remaining pellets in the bottles were weighed, and this weight was subtracted from the initial pellet weight before the start of the burrowing. To minimise the impact of microenvironmental conditions on burrowing performance, the individual cages were rotated on the shelves for each measurement.

The mean value of food burrowed during two basal activity measurements was considered the baseline burrowing activity for each animal. All subsequent experimental measurements were normalised to the basal activity (experimental measurement [%]/mean basal measurement [%]), and the obtained relative values were used for statistical analysis.

Burrowing behaviour was evaluated in three separate experiments. The first experiment (CYP+CBD=6, CYP+veh=6) was considered preliminary, as some changes were made to the burrowing protocol for the subsequent experiments; therefore, the results of this experiment were not included in the analysis. Eighteen animals were included in experiments 2 and 3 (CYP+CBD=9, CYP+veh=9). Of these, one animal (from the CYP+CBD group) failed to exhibit any burrowing activity during one of the experimental measurements and was therefore excluded from the analysis.
